# Nerve Surgery to Treat Intractable Genitofemoral Neuropathic Pain following Laparoscopic Live Kidney Donation

**DOI:** 10.1155/2018/9326975

**Published:** 2018-06-06

**Authors:** K. Ramdhani, M. J. A. Malessy, M. J. G. Simon, V. A. L. Huurman

**Affiliations:** ^1^Department of Transplant Surgery, Leiden University Medical Center, Leiden, Netherlands; ^2^Department of Neurosurgery, Leiden University Medical Center, Leiden, Netherlands; ^3^Department of Anesthesiology, Leiden University Medical Center, Leiden, Netherlands

## Abstract

To date live laparoscopic donor nephrectomies (LLDN) are frequently performed. The most common complications entail bleeding, wound infection, and incisional hernia. Here we discuss a 50-year-old patient with a severe less known complication, namely, postoperative persistent neuropathic pain in the scrotum and left upper leg. Satisfactory pain control could not be obtained in 3 years of postoperative pain treatment which consisted of neuroleptic drugs, blocks of the L1/L2 dorsal roots with local anaesthetics, and pulsed radiofrequency lesioning. Exploratory laparoscopy was performed to assess the aspect of the genitofemoral nerve (GFN). A hemoclip used for the closure of the ureter at the time of nephrectomy was found in close relation to the GFN. The clip was removed and the GFN was subsequently cut proximal to the side of this clip. Soon after surgery the patient was completely pain-free and could return to his normal activities. Surgery should be considered in case of GFN neuropathic pain following LLDN.

## 1. Introduction

Genitofemoral nerve (GFN) neuropathic pain after live laparoscopic donor nephrectomy is a rare complication which can cause pain and great discomfort [1]. The incidence of this complication is unknown. It is well recognized, however, that surgical procedures can cause neuropathic pain due to a nerve lesion or nerve entrapment. An example is ilioinguinal neuropathy following open inguinal hernia repair, for which an incidence of 11% is reported [2]. One-third of these patients experience limitations in daily leisure activities [2]. Treatment of GFN neuropathic pain following laparoscopic donor nephrectomy lacks clear guidelines or instructions for clinical practice [1]. Here we report the surgical treatment of persisting pain in the GFN innervating area, which was otherwise untreatable, several years after live laparoscopic donor nephrectomy.

## 2. Case Report

**Figure 1:**
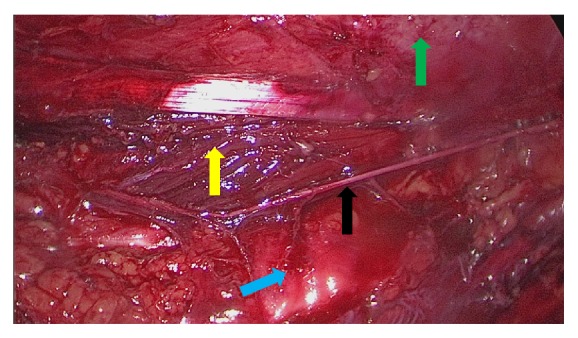
Perioperative view of the dissected proximal left genitofemoral nerve (GFN), just before neurectomy. Patient in right lateral decubitus position. Black arrow: GFN. Yellow arrow: musculus psoas. Green arrow: lateral abdominal wall. Blue arrow: intestine.

A 50-year-old man underwent live laparoscopic donor nephrectomy (LLDN), through transperitoneal approach, on his left kidney as a donor for his sister. This procedure went uneventful and the patient was discharged 3 days after surgery. However, within one week after the procedure the patient experienced pain at the left side of the scrotum and left upper leg. During physical examination, a hypersensitive scrotum (allodynia) was noted whereas no other abnormalities were seen. The urologist was consulted but no urologic complications were found. The neuropathic pain area befitted the genitofemoral nerve (GFN). Conservative treatment was initiated with neuroleptic drugs and blocks of the L1/L2 dorsal roots with local anaesthetics and pulsed radiofrequency lesioning. These treatments did not result in significant pain relief and he could not work. Three years after the start of the neuropathic pain, operative treatment aiming at pain relief was initiated. In a multidisciplinary meeting it was concluded that a nerve entrapment of the GFN was suspected to be the most likely cause of the patient's neuropathic pain. Eventually, an exploratory transperitoneal laparoscopy was performed. After extensive adhesiolysis, a hemoclip used for closure of the ureter at the time of nephrectomy was identified in close relation to the GFN and removed. Due to the local scarring it could not be surgically assessed to which extent the GFN was actually damaged or a neuroma was formed. It seemed, however, highly likely that the pain originated from the GFN in this area. We therefore cut the GFN proximally in a normal looking segment (Figure 1). Pathological analysis of the tissue confirmed this to contain nerve tissue. Within 2 weeks following surgery, the patient was completely pain-free and could return to his normal activities.

## 3. Discussion

Neuropathic pain in the area of the GFN as a complication after laparoscopic donor nephrectomy is rarely reported [3]. We could only find one case report similar to ours [1]. Although GFN damage as a cause is rarely reported, orchialgia as a symptom is a common finding [4–6]. Postoperative neuropathic pain is well documented in Lichtenstein procedures for inguinal hernia repair. Approximately 11 percent have postherniorrhaphy inguinodynia after inguinal hernia repair [1]. The pain is often deemed of neuropathic (nerve-related) origin. Neurectomy of the ilioinguinal nerve, occasionally in combination with iliohypogastric or genitofemoral nerve resection, is frequently considered. Multiple studies show that selective neurectomy decreases postherniorrhaphy inguinodynia [4, 7]. Our case shows that careful stapling of the ureter especially when in close relationship to the GFN is of paramount importance. In such cases we advocate surgery if conservative therapy fails. To assure successful treatment, cutting the nerve might be the best option rather than mere removal of the clip. In general, GFN damage or entrapment should be included in the differential diagnosis of persistent scrotum pain following laparoscopic donor nephrectomy.
